# Inclusion of diabetic retinopathy screening strategies in national-level diabetes care planning in low- and middle-income countries: a scoping review

**DOI:** 10.1186/s12961-022-00940-0

**Published:** 2023-01-02

**Authors:** Katie Curran, Prabhath Piyasena, Nathan Congdon, Lisa Duke, Belma Malanda, Tunde Peto

**Affiliations:** 1 Centre for Public Health, Institute of Clinical Science, Queen’s University Belfast, Block A, Royal Victoria Hospital, Belfast, BT12 6BA Northern Ireland UK; 2 Directorate of Policy Analysis and Development, Ministry of Health, Columbo, Sri Lanka; 3 Zhongshan Ophthalmic Centre, Sun Yat-Sen University, Guangzhou, China; 4 Orbis International, New York, USA; 5 International Diabetes Federation, Brussels, Belgium; 6 Department of Ophthalmology, Belfast Health and Social Care Trust, Belfast, UK

**Keywords:** Diabetic retinopathy, Policies, Diabetic eye screening programmes

## Abstract

**Background:**

Diabetes is a major public health concern, with approximately 80% of the burden falling on low- and middle-income countries (LMICs). Diabetic retinopathy (DR) is one of the most common microvascular complications of diabetes, and early detection through diabetic eye screening programmes is essential to prevent visual impairment and blindness. Careful planning at a national level is crucial for effective implementation of such programmes.

**Methods:**

A scoping review was conducted, and the protocol was published previously to explain the methods in detail. Data were collected from databases and searches, including grey literature. Furthermore, consultations were conducted with key informants from LMICs.

**Results:**

Lower-middle-income countries (29/50, 58%) and upper-middle-income countries (27/59, 45.8%) are making more progress than low-income countries (4/29, 13.8%) in terms of DR policy planning. However, no identified data for published policies have actually implemented national DR policies. Compared to low-income and lower-middle-income countries, upper-middle-income countries are making the most progress in implementing national diabetic eye screening programmes; however, their progress is still slow, with only 5/59 (8.5%) having such programmes.

**Conclusion:**

There are significant gaps in the literature, with no data reported for 78/138 (56.5%) LMICs. Further research is clearly needed to support and document DR policy development in LMICs.

**Supplementary Information:**

The online version contains supplementary material available at 10.1186/s12961-022-00940-0.

## Background

Globally, 463 million people are estimated to have diabetes mellitus (DM), with more than 80% living in low- and middle-income countries (LMICs) [1]. Diabetic retinopathy (DR) is one of the most common microvascular complications of DM, and early detection through DR screening and timely treatment can prevent visual impairment and blindness [2, 3]. DR is listed as a priority eye disease in the 2030 IN SIGHT strategy; however, many countries are faced with challenges in adopting public health approaches to manage this condition [4]. The IN SIGHT strategy has been developed with the aim of eliminating avoidable blindness and targeting the world’s leading causes of avoidable visual impairment by 2030 [4]. DR is not typically included in health sector strategic plans, especially in LMICs [5]. The lack of integration of DR services in health sector strategic plans means that it has been excluded from the national planning and budgeting for services in the past [5]. A lack of clearly defined care pathways can make it more challenging to define DR policies, or design and implement screening programmes. Despite the growing attention to addressing the issue of DR, many important policy questions remain unanswered in LMICs. There is a general lack of attention on the need to improve DR care by policy-makers and a lack of advocacy [6].

National health policies, strategies and plans provide a framework for countries to deal with public health issues, particularly those related to the Sustainable Development Goals (SDGs) and to other national priority health problems, such as noncommunicable diseases (NCDs). Good collaboration between stakeholders for developing policies, strategies and plans leads to a more balanced and coherent approach, and better use of resources for health [7]. Careful DM and DR planning at a national level is crucial for effective implementation, and it is vital to consider the development, review and scrutiny of policy and legislation. The extent to which DR is prioritized in national plans, strategies and policies is crucial for programme sustainability. Long-term funding is also essential to support services and allocate resources [8]. The aim of this scoping review was to identify countries who have not included DR in their national DM/NCD plan/policies, seeking to inform government bodies and leading national health services. The findings from this scoping review will support improved access to DM-related eye care and promote global health equity.

### Study objectives

- Identify LMICs that have not included DR services in their national DM strategic plans, action plans or policies, or as part of their NCD policies or prevention of blindness plans.
- To assess gaps in national-level DR services planning in LMICs.

## Methods

We employed a scoping review protocol that was published previously [9]. Methods for this scoping review were developed based on the Arksey and O'Malley methodological framework, and the more advanced framework of Levac et al. [10, 11]. Recommendations by the Joanna Briggs Institute were also applied to increase clarity [12]. The scoping review was conducted and reported in accordance with the Preferred Reporting Items for Systematic Reviews and Meta-Analyses extension for scoping reviews (PRISMA-ScR) guidelines (Additional file 1) [13].

### Data sources and search strategy

This scoping review is as comprehensive as possible in identifying data (published and unpublished) from October 1989 (St. Vincent Declaration—reduce diabetes-related blindness by at least one third) to February 2020. The primary sources used were MEDLINE (Ovid), Embase (Ovid), and the Cochrane Database of Systematic Reviews (CDSR) and the Cochrane Central Register of Controlled Trials (CENTRAL) in the Cochrane Library. Grey literature was obtained from reference lists of included articles. We also searched the WHO official sites and ministry of health (MOH) websites to determine whether policies or relevant documentation was available for all LMICs included in the scoping review (*n* = 137). We searched specifically for diabetes-related policy articles and retrieved 16 studies in total.

### Study selection

To be eligible for inclusion, the study/article had to be (1) conducted in LMICs to generate evidence to inform the development of national- or subnational-level DR screening and treatment programmes, (2) published articles/action plans/policy documents in LMICs on DM, NCD or DR that describe strategies for DR screening at the national or subnational-level or (3) published articles/reports/policy documents in LMICs on eye care that describe strategies for the prevention of blindness and visual impairment due to DR (4) published in English. The search focused solely on LMICs, to determine which countries have or are developing diabetic eye screening programmes (DESPs) for their populations with DM. The LMICs were selected by income level according to the World Bank lending group classification [14]. Two reviewers (KC, PP) independently screened titles and abstracts, cross-referencing the results. Titles and abstracts that did not meet the eligibility criteria were excluded, and full-text articles were retrieved for those that did meet the criteria.

### Data extraction

A Microsoft^®^ Excel database was generated to extract data from the full articles. Studies were selected according to the data extraction framework recommended by the Joanna Briggs Institute for scoping reviews (Additional file 2). One reviewer (KC) was responsible for extracting data from each study identified in the review, and these were verified by the co-reviewer (PP). To ensure good inter-rater agreement between the reviewers, a subset of the included articles (10%) were assessed. Any discrepancies were discussed by both reviewers until consensus was reached. If the reviewers could not agree on some studies, a third reviewer (the arbitrator) was available.

### Collating, summarizing and reporting the results

A policy cycle framework with four stages—agenda-setting (early and late phases), policy formulation, policy implementation and evaluation—was adopted to address gaps in national-level DR service planning, and a colour-coded system was used to highlight country progress in terms of their development in the policy cycle. A quantitative analysis was carried out to map the data in tabular form, highlighting country progress in terms of national DESP implementation. The most updated evidence for a country was used (Additional file 3). Full implementation means a national DESP is available, and partial implementation means a country is in the process of DR screening, but coverage is not at a national level.

Evaluating the evidence of DR care in the LMICs was facilitated by synthesizing findings on studies describing DR national plans, strategies, and policies and national DESPs. Countries were divided into themes according to the country income level and name, and gaps illustrate that no data was identified (see Additional file 3 for more detailed information). These themes were further divided into subthemes for each income level to demonstrate the countries development’ in the (1) policy cycle stage and (2) diabetic eye screening implementation stage.

### Consultations with key stakeholders

A consultation stage was included to add methodological rigour to the scoping review. The relevant stakeholder(s) including nongovernmental organizations (NGOs) and government officials were contacted to offer additional sources of information, perspectives and meaning to the scoping review. In addition, consultations were carried out with key informants from the International Diabetes Federation (IDF) to identify gaps in the results for each country, and to inform future research. Preliminary findings were provided to stakeholders to inform the consultation, and this allowed them to build on the evidence and offer a higher level of meaning and context to strengthen the preliminary findings. The key informants were contacted via our partnerships with the IDF. All results were aggregated, including those obtained from the IDF consultations.

## Results

**Fig. 1:**
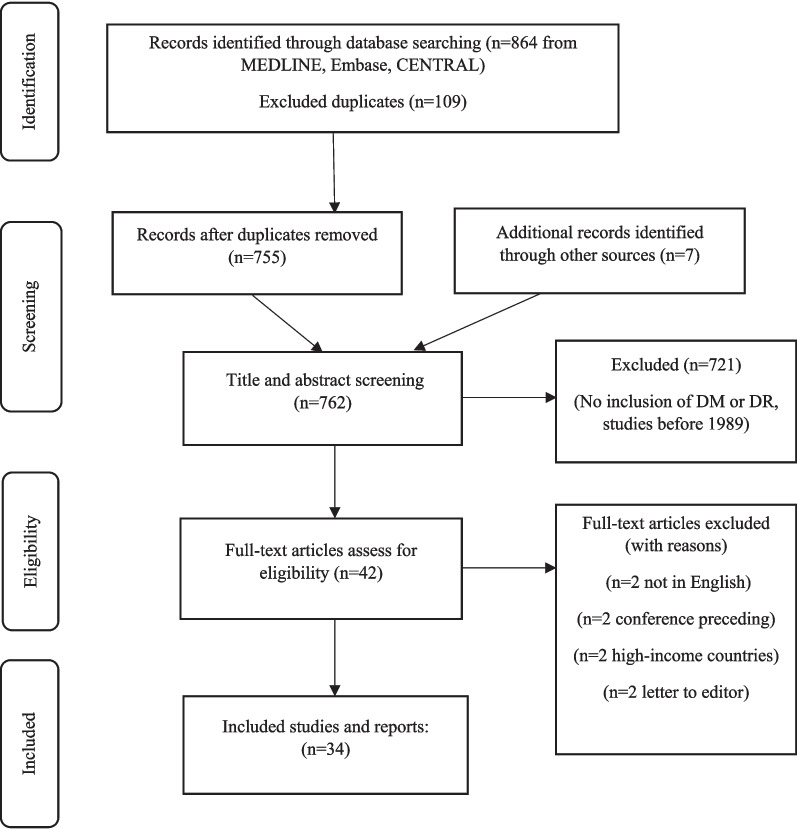
PRISMA flow chart of eligible studies for scoping review

The search identified 864 articles in total, and an additional seven records were identified from other sources (websites and bibliographies). Duplicates (109/864; 12.6%) were removed, and 762 titles and abstracts were reviewed for inclusion in the review. Based on the information provided in the titles and abstracts, 720 (94.5%) articles did not meet the inclusion criteria and were excluded. In total, 42 full-text articles were assessed for eligibility, and 8/42 (19.0%) were excluded for various reasons (Fig. 1). Only two non-English studies were excluded. Abstracts for both studies were retrieved in English and were excluded since they did not meet the inclusion criteria. Furthermore, data were collected for 36/138 (26.0%) LMICs during the consultation stage. In total, no data were identified for 78/138 (56.5%) LMICs in this scoping review.

### General characteristics of included studies

The included research studies and reports were published between 2006 and 2020 and comprised 32 published studies including cross-sectional studies (*n* = 20) [15–34], reviews (*n* = 4) [4, 35–37], cohort studies (*n* = 2) [38, 39], qualitative studies (*n* = 5) [40–44] and a cluster-randomized controlled trial (*n* = 1) [45], and two programme evaluations [8, 46]. All included documents were in English.

### DR national plans, strategies and policies in LMICs

No LMICs included in our scoping review are beyond stage 2 (DR policy formulation) of the policy cycle framework, and these countries are stratified according to country income level. Overall, reported data on DR planning and implementation in LMICs are scarce, and this is particularly noticeable in low-income countries (LICs).

#### LICs

Only four LICs (*n* = 4/29, 13.8%) were identified in this review, two of which were within the early stages of the agenda-setting DR policy cycle (situation analysis), and two within the later stage of the agenda-setting policy cycle. This review highlights that the majority of LICs (25/29 = 86.2%) have no identifiable data to gauge progress on DR policy planning (Table 1).

**Table 1:** Status of development of a national-level DR policy (based on the policy cycle approach) by country income level

Country income level	DR Policy cycle				
	Agenda-setting (*n*, %)	Policy formulation (*n*, %)	Policy implementation (*n*, %)	Evaluation (*n*, %)	Data not identified
Low-income (*n* = 29)	4 (13.8)	0 (0)	0 (0)	0 (0)	25 (86.2)
Lower-middle (*n* = 50)	21 (42)	8 (16.0)	0 (0)	0 (0)	21 (42.0)
Upper-middle (*n* = 59)	22 (37.3)	5 (8.5)	0 (0)	0 (0)	32 (54.2)

Policy cycle consists of four main stages: (1) agenda-setting, consisting of an early stage (situation analysis) and late stages (action plans), (2) policy formulation, (3) implementation and (4) evaluation

Countries are divided into income levels based on World Bank data (2020)

#### Lower-middle-income countries

In total, 21/50 (42%) lower-middle-income countries have reached the agenda-setting stage (stage 1) of the DR policy cycle (Table 1). Compared to LICs, lower-middle-income countries are making greater progress in tackling DR; however, 21/50 (42%) still have no identified data on DR planning. While lower-middle-income countries have made more progress than LICs and upper-middle-income countries (UMICs) in developing DR policies, still only 8/50 (16.0%) have reached the policy formulation stage of the policy cycle (Table 1).

#### UMICs

While UMICs have made more progress than poorer countries in terms of DR agenda-setting, still only about a third (22/59 = 37.3%) have done so, and only 5/59 (8.5%) UMICs have developed a DR policy (Table 1). Similar to lower-middle-income countries, data are available for a limited number of UMICs (27/59 = 45.8%) to gauge progress in DR planning.

### National DESPs

#### LICs

Only one LIC (1/29, 3.4%) reportedly has a partially implemented DESP (Haiti, IDF). For a high percentage of countries, data could not be identified (27/29, 93.1%) (Table 2).

**Table 2:** Status of development of national DESP in LMICs by country income level

Country income level	Stage of DESP development			
	No DESP (*n*, %)	Partially implemented DESP (*n*, %)	National DESP (*n*, %)	Data not identified
Low-income (*n* = 29)	1 (3.4)	1 (3.4)	0 (0)	27 (93.1)
Lower-middle (*n* = 50)	5 (10.0)	18 (36.0)	1 (2.0)	26 (52.0)
Upper-middle (*n* = 59)	5 (8.5)	11 (18.6)	5 (8.5)	38 (64.4)

Countries are divided into income levels based on World Bank data (2020)

DESPs implemented regionally mean those implemented in different regions within the country

#### Lower-middle-income countries

Only one lower-middle-income country (Republic of the Congo [IDF]) has a national DESP. In total, 18/50 (36.0%) lower-middle-income countries have DESPs which are implemented, but not yet with full national coverage, and 5/50 (10.0%) do not have any DESPs. Furthermore, 26/50 (52.0%) have no identified data (Table 2).

#### UMICs

Based on available data, a comparatively small number of UMICS (5/59 = 8.47%) are completely without DESPs. In total, 11/59 (18.6%) UMICs have partially implemented DESPs, and 5/59 (8.5%) do have fully implemented national DESPs with national coverage (Table 2).

#### Consultation versus publication data

There was overlap for seven countries between information collected from the consultation stage and the publications. The information between the included studies did not differ for Bangladesh, Mexico and Nigeria. India, Peru and Zambia differed in that the IDF reported no national programme, whereas publications reported that there was a subnational screening programme. For Argentina, no information was identified from the IDF representative, and the publication reported that no programme was available. Finally, the publication for Kenya reported that their policy was at the agenda-setting stage, while IDF stated that they had reached the policy formulation stage. Both resources stated that a DR programme was partially implemented (Table 3).

**Table 3:** Summary of LMICs with and without DM and DR plans, strategies or policies and existing national DESPs

Country income level	Country name	Status of development of a national-level DR policy (agenda-setting, policy formulation, policy implementation, evaluation)	Level of DESP implementation (no DESP, partially implemented, national DESP)
Low-income countries (*n* = 29)	Afghanistan	No data identified	No data identified
	Burkina Faso	No data identified	No data identified
	Burundi	No data identified	No data identified
	Central Africa	No data identified	No data identified
	Chad	No data identified	No data identified
	Congo, Democratic Republic of	No data identified	No data identified
	Eritrea	No data identified	No data identified
	Ethiopia	No data identified	No data identified
	Gambia	No data identified	No data identified
	Guinea	No data identified	No data identified
	Guinea-Bissau	No data identified	No data identified
	Haiti	Agenda-setting (early stage)	Partially implemented
	Korea, Democratic People’s Republic of	No data identified	No data identified
	Liberia	No data identified	No data identified
	Madagascar	No data identified	No data identified
	Malawi	Agenda-setting (later stage)	No data identified
	Mali	No data identified	No data identified
	Mozambique	No data identified	No data identified
	Niger	No data identified	No data identified
	Rwanda	No data identified	No data identified
	Sierra Leone	No data identified	No data identified
	Somalia	No data identified	No data identified
	South Sudan	No data identified	No data identified
	Sudan	No data identified	No data identified
	Syrian Arab Republic	No data identified	No data identified
	Tajikistan	No data identified	No data identified
	Togo	No data identified	No data identified
	Uganda	Agenda-setting (later stage)	No data identified
	Yemen, Republic of	Agenda-setting (later stage)	No DESP
Lower-middle-income countries (*n* = 50)	Angola	Agenda-setting (early stage)	Partially implemented
	Algeria	Agenda-setting (early stage)	Partially implemented
	Bangladesh	Agenda-setting (later stage)	Partially implemented
	Benin	No data identified	No data identified
	Bhutan	No data identified	No data identified
	Bolivia	No data identified	No data identified
	Cape Verde	Agenda-setting (later stage)	No data identified
	Cambodia	No data identified	No data identified
	Cameroon	Agenda-setting (early stage)	No DESP
	Comoros	No data identified	No data identified
	Congo, Republic of	Policy formulation	National DESP
	Côte d'Ivoire	No data identified	No data identified
	Djibouti	No data identified	No data identified
	Egypt	Agenda-setting (early stage)	No DESP
	El Salvador	No data identified	No data identified
	Eswatini	No data identified	No data identified
	Ghana	Agenda-setting (later stage)	No data identified
	Honduras	Agenda-setting (early stage)	Partially implemented
	India	Agenda-setting (early stage)	Partially implemented
	Kenya	Policy formulation	Partially implemented
	Kiribati	Policy formulation	No DESP
	Kyrgyzstan (Kyrgyz Republic)	Agenda-setting (early stage)	No DESP
	Lao People’s Democratic Republic (Laos)	No data identified	No data identified
	Lesotho	Agenda-setting (early stage)	Partially implemented
	Mauritania	No data identified	No data identified
	Micronesia, Federated States of	No data identified	No data identified
	Moldova	No data identified	No data identified
	Mongolia	Agenda-setting (early stage)	Partially implemented
	Morocco	No data identified	No data identified
	Myanmar	No data identified	No data identified
	Nepal	Agenda-setting (early stage)	Partially implemented
	Nicaragua	No data identified	No data identified
	Nigeria	Agenda-setting (later stage)	No data identified
	Pakistan	Agenda-setting (early stage)	Partially implemented
	Papua New Guinea	Agenda-setting (early stage)	No data identified
	Philippines	Agenda-setting (early stage)	No DESP
	São Tomé and Principe	No data identified	No data identified
	Senegal	No data identified	No data identified
	Solomon Islands	Policy formulation	Partially implemented
	Sri Lanka	Agenda-setting (early stage)	Partially implemented
	Tanzania	Agenda-setting (later stage)	Partially implemented
	Timor-Leste	Agenda-setting (early stage)	No data identified
	Tunisia	Policy formulation	Partially implemented
	Ukraine	No data identified	No data identified
	Uzbekistan	Policy formulation	Partially implemented
	Vanuatu	Policy formulation	Partially implemented
	Vietnam	Agenda-setting (early stage)	Partially implemented
	West Bank and Gaza (Palestine)	Policy formulation	Partially implemented
	Zambia	Agenda-setting (early stage)	Partially implemented
	Zimbabwe	No data identified	No data identified
Upper-middle-income countries (*n* = 58)	Albania	Agenda-setting (early stage)	No DESP
	American Samoa	No data identified	No data identified
	Argentina	Agenda-setting (early stage)	No DESP
	Armenia	No data identified	No data identified
	Azerbaijan	No data identified	No data identified
	Belarus	No data identified	No data identified
	Belize	Agenda-setting (early stage)	No DESP
	Bosnia and Herzegovina	Agenda-setting (early stage)	Partially implemented
	Botswana	No data identified	National DESP
	Brazil	Policy formulation	National DESP
	Bulgaria	No data identified	No data identified
	China	Agenda-setting (early stage)	Partially implemented
	Colombia	No data identified	No data identified
	Costa Rica	Agenda-setting (later stage)	No data identified
	Cuba	No data identified	No data identified
	Dominica	No data identified	No data identified
	Dominican Republic	No data identified	No data identified
	Equatorial Guinea	No data identified	No data identified
	Ecuador	Agenda-setting (early stage)	Partially implemented
	Fiji	Agenda-setting (early stage)	Partially implemented
	Gabon	No data identified	No data identified
	Georgia	No data identified	No data identified
	Grenada	No data identified	No data identified
	Guatemala	Agenda-setting (early stage)	No data identified
	Guyana	No data identified	No data identified
	Indonesia	No data identified	No data identified
	Iran, Islamic Republic of	Agenda-setting (early stage)	No data identified
	Iraq	Agenda-setting (early stage)	No DESP
	Jamaica	Agenda-setting (later stage)	No data identified
	Jordan	No data identified	No data identified
	Kazakhstan	No data identified	No data identified
	Kosovo	No data identified	No data identified
	Lebanon	Agenda-setting (early stage)	Partially implemented
	Libya	No data identified	No data identified
	Malaysia	Policy information	National DESP
	Maldives	No data identified	No data identified
	Marshall Islands	No data identified	No data identified
	Mauritius	Agenda-setting (early stage)	National DESP
	Mexico	Agenda-setting (early stage)	No DESP
	Montenegro	No data identified	No data identified
	Namibia	No data identified	No data identified
	North Macedonia	Policy formulation	Partially implemented
	Nauru	No data identified	No data identified
	Paraguay	No data identified	No data identified
	Peru	Agenda-setting (early stage)	Partially implemented
	Romania	No data identified	No data identified
	Russian Federation	No data identified	No data identified
	Samoa	Agenda-setting (early stage)	Partially implemented
	Serbia	Policy information	National DESP
	South Africa	Agenda-setting (early stage)	Partially implemented
	St. Lucia	Agenda-setting (early stage)	Partially implemented
	St. Vincent and the Grenadines	No data identified	No data identified
	Suriname	No data identified	No data identified
	Thailand	Policy formulation	Partially implemented
	Tonga	Agenda-setting (early stage)	No data identified
	Turkey	Agenda-setting (early stage)	No data identified
	Turkmenistan	No data identified	No data identified
	Tuvalu	No data identified	No data identified
	Venezuela, Bolivarian Republic of	No data identified	No data identified

## Discussion

Data regarding national DR policies or national DESP planning in LMICs are scarce, as highlighted in the current scoping review. The highest burden of DM is concentrated in LMICs, and LICs have made the least progress in terms of DR planning and implementation. No LICs have national DESPs, and this is followed by lower-middle-income countries. UMICs have made the most progress in terms of national DESP implementation. Many LMICs have not yet reached the agenda-setting stage of a DR policy cycle; thus, there is a need for situational analyses to determine the prevalence of DR and estimate the screening burden in these LMICs. This will allow planning for policies, health services and human resource development, providing a baseline to monitor future trends. The prevalence of DR in the context of each country must be considered when training and distributing eye care personnel. Globally, the gap is widening between the need for eye health workers to provide essential services and the availability and government capacity to employ these workers [48, 49]. This is particularly true in LMICs compared to high-income countries (HICs) [49].

In HICs, DR screening is mostly conducted through national systematic programmes, whereas LMICs are unlikely to have population-based screening. In HICs, digital retinal photography is often used, and fundus images are graded by trained eye care personnel. People with signs of sight-threatening DR are referred for clinical assessment or management at the tertiary level [50]. Contrastingly, LMICs provide DR screening on an opportunistic level, and there are acute shortages in eye care personnel. Lack of funding and implementation of relevant services are major barriers to DR screening in LMICs [48, 51]. Malaysia developed a diabetic eye registry between 2007 and 2008 and has support for DR screening from their MOH. People with DM are screened by ophthalmologists in health clinics and most hospitals. Although Malaysia is making progress in terms of DESP implementation, they too have challenges with managing DR. These include lack of awareness of DR, lack of skilled personnel to detect DR, and screening only reaching a small proportion of the population [52]. Similarly, Mauritius developed a 10-year plan for DM in the form of a DM national service framework and established their objectives through partnerships with the Mauritius Institute of Health [53]. Importantly, they had support from the MOH and Quality of Life of Mauritius [53].

The first LMIC in the world to launch a national programme for control of blindness was India in 1976 [54]. India is making relatively good progress in terms of DR planning, and this is important, as India has the highest total burden of DM among LMICs [54]. In terms of DR planning, UMICs are performing best; however, barriers still exist. Shortages in human resources and infrastructure are common problems in these countries as well. Poor information and auditing systems are often challenging for DR screening services. In LMICs, ophthalmic teams may have prepared DR policies and put them forward as part of an NCD policy (as a strategic objective). This means that a whole DR policy is available under the national NCD policy as one strategy. This commonly occurs in LMICs versus HICs. In a HIC, such as England, assessment and treatment facilities for DR are available as part of the National Health Service [50]. In LMICs, lack of collaboration between the health sector and other key stakeholders appears to be ubiquitous in developing policy documents [55]. Reliance on evidence to support decisions and present essential actions is crucial for comprehensive, consistent and sustainable policies [55]. Poor availability and accessibility of research are often considered major barriers to policy-makers’ use of research [56]. Collecting evidence, considering the local needs and resources, and examining the effectiveness of past efforts are essential steps before investing in new DR policies [49].

### Strengths

This is the first scoping review to explore national-level DR planning in LMICs, in the context of country income level. This review has identified gaps in the existing literature, which is particularly important as it highlights which LMICs require additional DR support and development for the future. Action needs to be taken to address the lack of DESPs, and research describing and evaluating these is also required. An iterative team approach was utilized to select relevant studies and extract data. The study successfully explored the organization and development of DR screening in national-level DM care planning in LMICs. Countries that lack national-level DR planning have been identified, which is beneficial for funders and programme planners. The findings from this study may strengthen policy and research in LMICs. Finally, the study adopted a consultation stage in the methodology to strengthen the scoping review results.

### Limitations

The lack of good-quality, publicly available policy-related documents from LMICs was a major challenge in this scoping review. Despite the researchers' best efforts, it is inevitable that relevant evidence may have been missed, especially due to the limits of the English-language searches. This decision was made with the research team due to limited resources for translation. For these reasons, we may be underreporting actual progress in LMICs. To address the lack of available data, consultations were conducted with country representatives to provide more clarity to our results, although lack of comprehensive knowledge and awareness regarding DR policies may have led to information bias. The response rate during the consultation stage of this scoping review was low (36/138; 26.0%), which may not be fully representative of all LMICs. Our search did not capture information about the Mauritius National Service Framework for DM, and although their primary focus area was DM foot care, the framework includes national diabetic eye screening. This information was retrieved from the World Diabetes Foundation website and may have had added value to the review. Reviewing NGO websites in the future could help to obtain additional data. Furthermore, building relationships and communicating regularly with relevant country-specific partners is recommended in order to capture relevant data. Finally, an information specialist helped to develop the extensive search strategy required to conduct the scoping; however, we recognize that the inclusion of “evaluation” and policy cycle terms, namely, agenda-setting, in the search criteria may have allowed us to capture more evaluation studies.

### Implications for future research

Establishing appropriately funded national-level policies or plans that target the impact of DM and DR is likely to be advantageous when coupled with adequate resource allocation, support and effective leadership. While addressing DR is not a simple task, contributions from all key stakeholders (governments, healthcare providers, people with DM and societies) could potentially reduce the burden of the disease on the individual, their carers and society. Based on the results of this scoping review, further research and advocacy work is required to achieve the intended impact, so that MOHs can implement strategies and policies to improve access to eye care for people with DM. This scoping review has provided a baseline for follow-up studies to track progress.

The Diabetic Retinopathy Network (DR-NET) has made an impressive start to addressing the burden of DR in low- and middle-income commonwealth countries in Africa, the Caribbean and the Pacific Islands [57]. These partnerships are allowing countries to build capacity for DR and share learning experiences. Further developments are needed to help additional LMICs across the globe.

## Conclusions

The current study highlighted significant gaps in the literature where no data were reported for many LMICs. Building a prioritized research agenda of the recent findings is a crucial next step towards catalysing these necessary improvements within and across LMICs to address the current and emerging challenges of DM and DR. Further research is clearly needed to develop a body of evidence that is adequate to support effective service and DR policy development in LMICs. International agencies and national governments should take a leadership role in developing and implementing comprehensive policies that make DR prevention a global and national policy.

## Supplementary Information

**Additional file 1.** PRISMA-ScR Checklist.

**Additional file 2.** Search strategy.

**Additional file 3.** Low and middle-income countries with and without diabetes and diabetic retinopathy plans, strategies or policies and existing National diabetic eye screening programmes.

## Data Availability

The data generated or analysed during this study are included in this published article [and its additional files].

## Abbreviations

DM: Diabetes mellitus
DR: Diabetic retinopathy
DESP: Diabetic eye screening programme
LMICs: Low- and middle-income countries
LICs: Low-income countries
UMICs: Upper-middle–income countries
HICs: High-income countries
MOH: Ministries of health
