# Supplementary material for: Inclusion of diabetic retinopathy screening strategies in national-level diabetes care planning in low- and middle-income countries: a scoping review
Source: Health Res Policy Syst. 2023 Jan 2;21:2. doi: 10.1186/s12961-022-00940-0 (PMC9808973; doi:10.1186/s12961-022-00940-0)
Supplement: Supplementary file 2 — Additional file 2. Search strategy. [file 12961_2022_940_MOESM2_ESM.docx]

**Additional file 2: Search strategy**

| **MEDLINE/Ovid** | **EMBASE/Ovid** | **Cochrane Library** |
| --- | --- | --- |
| 1. exp diabetes mellitus/ or diabetes mellitus, experimental/ or diabetes mellitus, type 1/ or wolfram syndrome/ or diabetes mellitus, type 2/ or diabetes mellitus, lipoatrophic/ or diabetes, gestational/ or diabetic ketoacidosis/ or donohue syndrome/ or latent autoimmune diabetes in adults/ or prediabetic state/ 2. Diabetic Retinopathy/ 3. Retinopathy.mp. 4. Macular Edema/ 5. Exp Eye Diseases/ 6. 2 or 3 or 4 or 5 7. 1 and 6 8. Mass Screening/ 9. Vision tests/or vision screening/ or visual acuity/ 10. Photography/ 11. Fundus image.mp. 12. Ophthalmoscopes/or retinoscopes/ or slit lamp/ 13. Ophthalmoscopy/ or retinoscopy/ 14. Fundoscopy.mp. 15. 8 or 9 or 10 or 11 or 12 or 13 or 14 16. 7 and 15 17. Developing Countries/ 18. cambodia/ or indonesia/ or myanmar/ or philippines/ or thailand/ or timor-leste/ or vietnam/ or bangladesh/ or bhutan/ or india/ or afghanistan/ or iran/ or iraq/ or turkey/ or yemen/ or nepal/ or pakistan/ or sri lanka/ 19. africa/ or africa, northern/ or algeria/ or egypt/ or libya/ or morocco/ or tunisia/ or "africa south of the sahara"/ or africa, central/ or cameroon/ or central african republic/ or chad/ or congo/ or "democratic republic of the congo"/ or equatorial guinea/ or gabon/ or "sao tome and principe"/ or africa, eastern/ or burundi/ or djibouti/ or eritrea/ or ethiopia/ or kenya/ or rwanda/ or somalia/ or south sudan/ or sudan/ or tanzania/ or uganda/ or africa, southern/ or angola/ or botswana/ or eswatini/ or lesotho/ or malawi/ or mozambique/ or namibia/ or south africa/ or zambia/ or zimbabwe/ or africa, western/ or benin/ or burkina faso/ or cabo verde/ or cote d'ivoire/ or gambia/ or ghana/ or guinea/ or guinea-bissau/ or liberia/ or mali/ or mauritania/ or niger/ or nigeria/ or senegal/ or sierra leone/ or togo/ 20. cuba/ or dominican republic/ or haiti/ or jamaica/ or belize/ or costa rica/ or el salvador/ or guatemala/ or honduras/ or nicaragua/ or mexico/ 21. "democratic people's republic of korea"/ or mongolia/ 22. comoros/ or madagascar/ or mauritius/ or sri lanka/ 23. asia, central/ or kazakhstan/ or kyrgyzstan/ or tajikistan/ or turkmenistan/ or uzbekistan/ 24. argentina/ or bolivia/ or brazil/ or colombia/ or ecuador/ or paraguay/ or peru/ or suriname/ or venezuela/ 25. Syria/ 26. Kiribati.mp. 27. Laos/ 28. Micronesia/ or samoa/ or tonga/ 29. albania/ or "bosnia and herzegovina"/ or bulgaria/ or kosovo/ or moldova/ or montenegro/ or romania/ or russia/ or serbia/ or ukraine/ 30. melanesia/ or fiji/ or vanuatu/ 31. (West Bank and Gaza).mp. 32. armenia/ or azerbaijan/ 33. "Republic of Belarus"/ 34. dominica/ or saint lucia/ or "saint vincent and the grenadines"/ 35. China/ 36. “Georgia (Republic)”/ 37. Grenada/ 38. Jordan/ 39. Lebanon/ 40. Marshall Islands.mp. 41. “Republic of North Macedonia”/ 42. Nauru.mp. or Micronesia/ 43. 17 or 18 or 19 or 20 or 21 or 22 or 23 or 24 or 25 or 26 or 27 or 28 or 29 or 30 or 31 or 32 or 33 or 34 or 35 or 36 or 37 or 38 or 39 or 40 or 41 or 42 44. 16 and 43 | 1. exp diabetes mellitus/ or diabetes mellitus, experimental/ or diabetes mellitus, type 1/ or wolfram syndrome/ or diabetes mellitus, type 2/ or diabetes mellitus, lipoatrophic/ or diabetes, gestational/ or diabetic ketoacidosis/ or donohue syndrome/ or latent autoimmune diabetes in adults/ or prediabetic state/ 2. Diabetic Retinopathy/ 3. Retinopathy.mp. 4. Macular Edema/ 5. Exp Eye Diseases/ 6. 2 or 3 or 4 or 5 7. 1 and 6 8. Mass Screening/ 9. Vision tests/or vision screening/ or visual acuity/ 10. Photography/ 11. Fundus image.mp. 12. Ophthalmoscopes/or retinoscopes/ or slit lamp/ 13. Ophthalmoscopy/ or retinoscopy/ 14. Fundoscopy.mp. 15. 8 or 9 or 10 or 11 or 12 or 13 or 14 16. 7 and 15 17. Developing Countries/ 18. cambodia/ or indonesia/ or myanmar/ or philippines/ or thailand/ or timor-leste/ or vietnam/ or bangladesh/ or bhutan/ or india/ or afghanistan/ or iran/ or iraq/ or turkey/ or yemen/ or nepal/ or pakistan/ or sri lanka/ 19. africa/ or africa, northern/ or algeria/ or egypt/ or libya/ or morocco/ or tunisia/ or "africa south of the sahara"/ or africa, central/ or cameroon/ or central african republic/ or chad/ or congo/ or "democratic republic of the congo"/ or equatorial guinea/ or gabon/ or "sao tome and principe"/ or africa, eastern/ or burundi/ or djibouti/ or eritrea/ or ethiopia/ or kenya/ or rwanda/ or somalia/ or south sudan/ or sudan/ or tanzania/ or uganda/ or africa, southern/ or angola/ or botswana/ or eswatini/ or lesotho/ or malawi/ or mozambique/ or namibia/ or south africa/ or zambia/ or zimbabwe/ or africa, western/ or benin/ or burkina faso/ or cabo verde/ or cote d'ivoire/ or gambia/ or ghana/ or guinea/ or guinea-bissau/ or liberia/ or mali/ or mauritania/ or niger/ or nigeria/ or senegal/ or sierra leone/ or togo/ 20. cuba/ or dominican republic/ or haiti/ or jamaica/ or belize/ or costa rica/ or el salvador/ or guatemala/ or honduras/ or nicaragua/ or mexico/ 21. "democratic people's republic of korea"/ or mongolia/ 22. comoros/ or madagascar/ or mauritius/ or sri lanka/ 23. asia, central/ or kazakhstan/ or kyrgyzstan/ or tajikistan/ or turkmenistan/ or uzbekistan/ 24. argentina/ or bolivia/ or brazil/ or colombia/ or ecuador/ or paraguay/ or peru/ or suriname/ or venezuela/ 25. Syria/ 26. Kiribati.mp. 27. Laos/ 28. Micronesia/ or samoa/ or tonga/ 29. albania/ or "bosnia and herzegovina"/ or bulgaria/ or kosovo/ or moldova/ or montenegro/ or romania/ or russia/ or serbia/ or ukraine/ 30. melanesia/ or fiji/ or vanuatu/ 31. (West Bank and Gaza).mp. 32. armenia/ or azerbaijan/ 33. "Republic of Belarus"/ 34. dominica/ or saint lucia/ or "saint vincent and the grenadines"/ 35. China/ 36. “Georgia (Republic)”/ 37. Grenada/ 38. Jordan/ 39. Lebanon/ 40. Marshall Islands.mp. 41. “Republic of North Macedonia”/ 42. Nauru.mp. or Micronesia/ 43. 17 or 18 or 19 or 20 or 21 or 22 or 23 or 24 or 25 or 26 or 27 or 28 or 29 or 30 or 31 or 32 or 33 or 34 or 35 or 36 or 37 or 38 or 39 or 40 or 41 or 42 44. 16 and 43 | #1 MeSH descriptor [Diabetes mellitus] explode all trees  #2 MeSH descriptor [Diabetic Retinopathy] explode all trees  #3 MeSH descriptor [Macular Edema] explode all trees  #4 MeSH descriptor [Macular Degeneration] explode all trees  #5 MeSH descriptor [Eye Diseases] explode all trees  #6 #2 OR #3 OR #4 OR #5  #7 #1 AND #6  #8 MeSH descriptor [Mass Screening] explode all trees  #9 MeSH descriptor [Vision Tests] explode all trees  #10 MeSH descriptor [Photography] explode all trees  #11 Fundus image  #12 MeSH descriptor [Ophthalmoscopes] explode all trees  #13 MeSH descriptor [Ophthalmoscopy] explode all trees  #14 Fundoscopy  #15 #8 OR #9 OR #10 OR #12 OR #13 OR #14  #16 #7 AND #15  #17 MeSH descriptor [Developing Countries] explode all trees  #18 MeSH descriptor [Afghanistan] this term only  #19 MeSH descriptor [Benin] this term only  #20 MeSH descriptor [Burkina Faso] this term only  #21 MeSH descriptor [Burundi] this term only  #22 MeSH descriptor [Central African Republic] this term only  #23 MeSH descriptor [Chad] this term only  #24 MeSH descriptor [Democratic Republic of the Congo] this term only  #25 MeSH descriptor [Eritrea] this term only  #26 MeSH descriptor [Ethiopia] this term only  #27 MeSH descriptor [Gambia] this term only  #28 MeSH descriptor [Guinea] this term only  #29 MeSH descriptor [Guinea-Bissau] this term only  #30 MeSH descriptor [Haiti] this term only  #31 MeSH descriptor [Democratic People’s Republic of Korea] this term only  #32 MeSH descriptor [Liberia] this term only  #33 MeSH descriptor [Madagascar] this term only  #34 MeSH descriptor [Malawi] this term only  #35 MeSH descriptor [Mali] this term only  #36 MeSH descriptor [Mozambique] this term only  #37 MeSH descriptor [Nepal] this term only  #38 MeSH descriptor [Niger] this term only  #39 MeSH descriptor [Rwanda] this term only  #40 MeSH descriptor [Sierra Leone] this term only  #41 MeSH descriptor [Somalia] this term only  #42 MeSH descriptor [South Sudan] this term only  #43 MeSH descriptor [Syria] this term only  #44 MeSH descriptor [Tajikistan] this term only  #45 MeSH descriptor [Tanzania] this term only  #46 MeSH descriptor [Togo] this term only  #47 MeSH descriptor [Uganda] this term only  #48 MeSH descriptor [Yemen] this term only  #49 MeSH descriptor [Angola] this term only  #50 MeSH descriptor [Bangladesh] this term only  #51 MeSH descriptor [Bhutan] this term only  #52 MeSH descriptor [Bolivia] this term only  #53 MeSH descriptor [Cabo Verde] this term only  #54 MeSH descriptor [Cambodia] this term only  #55 MeSH descriptor [Cameroon] this term only  #56 MeSH descriptor [Comoros] this term only  #57 MeSH descriptor [Congo] this term only  #58 MeSH descriptor [Cote d’lvoire] this term only  #59 MeSH descriptor [Djibouti] this term only  #60 MeSH descriptor [Egypt] this term only  #61 MeSH descriptor [El Salvador] this term only  #62 MeSH descriptor [Eswatini] this term only  #63 MeSH descriptor [Ghana] this term only  #64 MeSH descriptor [Honduras] this term only  #65 MeSH descriptor [India] this term only  #66 MeSH descriptor [Indonesia] this term only  #67 MeSH descriptor [Kenya] this term only  #68 MeSH descriptor [Micronesia] this term only  #69 Kiribati  #70 MeSH descriptor [Kyrgyzstan] this term only  #71 MeSH descriptor [Laos] this term only  #72 MeSH descriptor [Lesotho] this term only  #73 MeSH descriptor [Mauritania] this term only  #74 MeSH descriptor [Moldova] this term only  #75 MeSH descriptor [Mongolia] this term only  #76 MeSH descriptor [Morocco] this term only  #77 MeSH descriptor [Myanmar] this term only  #78 MeSH descriptor [Nicaragua] this term only  #79 MeSH descriptor [Nigeria] this term only  #80 MeSH descriptor [Pakistan] this term only  #81 MeSH descriptor [Papua New Guinea] this term only  #82 MeSH descriptor [Philippines] this term only  #83 MeSH descriptor [Sao Tome and Principe] this term only  #84 MeSH descriptor [Senegal] this term only  #86 MeSH descriptor [Melanesia] this term only  #87 MeSH descriptor [Sudan] this term only  #88 MeSH descriptor [Tunisia] this term only  #89 MeSH descriptor [Ukraine] this term only  #90 MeSH descriptor [Uzekistan] this term only  #91 MeSH descriptor [Vanuatu] this term only  #92 MeSH descriptor [Vietnam] this term only  #93 West Bank and Gaza  #94 MeSH descriptor [Zambia] this term only  #95 MeSH descriptor [Zimbabwe] this term only  #96 MeSH descriptor [Albania] this term only  #97 MeSH descriptor [Algeria] this term only  #98 MeSH descriptor [American Samoa] this term only  #99 MeSH descriptor [Argentina] this term only  #100 MeSH descriptor [Armenia] this term only  #101 MeSH descriptor [Azerbaijan] this term only  #102 MeSH descriptor [Republic of Belarus] this term only  #103 MeSH descriptor [Belize] this term only  #104 MeSH descriptor [Bosnia and Herzegovina] this term only  #105 MeSH descriptor [Botswana] this term only  #106 MeSH descriptor [Brazil] this term only  #107 MeSH descriptor [Bulgaria] this term only  #108 MeSH descriptor [China] this term only  #109 MeSH descriptor [Colombia] this term only  #110 MeSH descriptor [Costa Rica] this term only  #111 MeSH descriptor [Cuba] this term only  #112 MeSH descriptor [Dominica] this term only  #113 MeSH descriptor [Dominican Republic] this term only  #114 MeSH descriptor [Equatorial Guinea] this term only  #115 MeSH descriptor [Ecuador] this term only  #116 MeSH descriptor [Fiji] this term only  #117 MeSH descriptor [Gabon] this term only  #118 MeSH descriptor [Georgia (Republic)] this term only  #119 MeSH descriptor [Grenada] this term only  #120 MeSH descriptor [Guatemala] this term only  #121 MeSH descriptor [Guyana] this term only  #122 MeSH descriptor [Iran] this term only  #123 MeSH descriptor [Iraq] this term only  #124 MeSH descriptor [Jamaica] this term only  #125 MeSH descriptor [Jordan] this term only  #126 MeSH descriptor [Kazakhstan] this term only  #127 MeSH descriptor [Kosovo] this term only  #128 MeSH descriptor [Lebanon] this term only  #129 MeSH descriptor [Libya] this term only  #130 MeSH descriptor [Malaysia] this term only  #131 Maldives  #132 Marshall Islands  #133 MeSH descriptor [Mauritius] this term only  #134 MeSH descriptor [Mexico] this term only  #135 MeSH descriptor [Montenegro] this term only  #136 MeSH descriptor [Namibia] this term only  #137 Naura  #138 MeSH descriptor [Republic of North Macedonia] this term only  #139 Paraguay  #140 MeSH descriptor [Peru] this term only  #141 MeSH descriptor [Romania] this term only  #142 Russian Federation  #143 MeSH descriptor [Samoa] this term only  #144 MeSH descriptor [Serbia] this term only  #145 MeSH descriptor [Sri Lanka] this term only  #146 MeSH descriptor [South Africa] this term only  #147 MeSH descriptor [Saint Lucia] this term only  #148 St. Lucia  #149 MeSH descriptor [Saint Vincent and Grenadines] this term only  #150 Saint Vincent and Grenadines  #151 MeSH descriptor [Suriname] this term only  #152 MeSH descriptor [Thailand] this term only  #153 MeSH descriptor [Tonga] term only  #154 MeSH descriptor [Turkey] this term only  #155 MeSH descriptor [Turkmenistan] this term only  #156 Tuvalu  #157 MeSH descriptor [Venezuela] this term only  #158 #17 OR #18 OR #19 OR #20 OR #21 OR #22 OR #23 OR #24 #27 OR #28 OR #29 OR #30 OR #31 OR #32 OR #33 OR #34 OR #35 OR #36 OR #37 OR #38 OR #39 OR #40 OR #41 OR #42 OR #43 OR #44 OR #45 OR #46 OR #47 OR #48 OR #49 OR #50 OR #51 OR #52 OR #53 OR #54 OR #55 OR #56 OR #57 OR #58 OR #59 #OR #60 OR #61 OR #62 OR #63 OR #64 OR #65 OR #66 OR #67 OR #68 OR #69 OR #70 OR #71 OR #72 OR #73 OR #74 OR #75 OR #76 OR #77 OR #78 OR #79 OR #80 OR #81 OR #82 OR #83 OR #84 OR #85 OR #86 OR #87 OR #88 OR #89 OR #90 OR #91 OR #92 OR #93 OR #94 OR #95 OR #96 OR #97 #OR #98 OR #99 OR #100 OR #101 OR #102 OR #103 OR #104 OR #105 OR #106 OR #107 OR #108 OR #109 OR #110 OR #111 OR #112 OR #113 OR #114 OR #115 OR #116 OR #117 OR #118 OR #119 OR #120 OR #121 OR #122 OR #123 OR #124 OR #125 OR #126 OR #127 OR #128 OR #129 OR #130 OR #131 OR #132 OR #133 OR #134 OR #135 OR #136 OR #137 OR #138 OR #139 OR #140 OR #141 OR #142 OR #143 OR #144 OR #145 OR #146 OR #147 OR #148 OR #149 OR #150 OR #151 OR #152 OR #153 OR #154 OR #155 OR #156 OR #157  #159 #16 AND #158 |
| Returned searches without adding LMICs = 6007  Returned searches with LMICs = 269 | Returned searches without adding LMICs = 12113  Returned searches with LMICs = 587 | Returned searches without adding LMICs = 592  Returned searches with LMICs = 8 |
