# Supplementary material for: Inclusion of diabetic retinopathy screening strategies in national-level diabetes care planning in low- and middle-income countries: a scoping review
Source: Health Res Policy Syst. 2023 Jan 2;21:2. doi: 10.1186/s12961-022-00940-0 (PMC9808973; doi:10.1186/s12961-022-00940-0)
Supplement: Supplementary file 3 — Additional file 3. Low and middle-income countries with and without diabetes and diabetic retinopathy plans, strategies or policies and existing National diabetic eye screening programmes. [file 12961_2022_940_MOESM3_ESM.docx]

**Additional file 3: Low and middle-income countries with and without diabetes and diabetic retinopathy plans, strategies or policies and existing National diabetic eye screening programmes**

| **Country income level** | **Country**  **Name** | **Author name and publication year** | **Availability of a national DM / NCD action plan / strategic plan / policy** | **Status of inclusion of DR in national DM / NCD action plan / strategic plan / policy** | **Status of inclusion of DR in national blindness prevention action plan / strategic plan/policy** | **Status of development of a national level DR* policy (based on policy cycle approach)** | **Proposed DR screening modality at the national level** | **Level of implementation of a national level or sub-national level DESP** |
| --- | --- | --- | --- | --- | --- | --- | --- | --- |
| **LIC**  **(Low-income countries)** (n=29) | Afghanistan | No data identified | | | | | | |
|  | Burkina Faso | No data identified | | | | | | |
|  | Burundi | No data identified | | | | | | |
|  | Central Africa | No data identified | | | | | | |
|  | Chad | No data identified | | | | | | |
|  | Congo Dem Rep | No data identified | | | | | | |
|  | Eritrea | No data identified | | | | | | |
|  | Ethiopia | No data identified | | | | | | |
|  | Gambia | No data identified | | | | | | |
|  | Guinea | No data identified | | | | | | |
|  | Guinea-Bissau | No data identified | | | | | | |
|  | Haiti | IDF, 2020 | No | N/A | No data identified | Agenda setting (Early stage) | Automated image grading | Partially implemented (Sub-national) |
|  | Korea, Dem | No data identified | | | | | | |
|  | Liberia | No data identified | | | | | | |
|  | Madagascar | No data identified | | | | | | |
|  | Malawi | CECH, 2019 (46) | No data identified | No data identified | National DR framework in place | Agenda setting (Later stage) | No data identified | No data identified |
|  | Mali | No data identified | | | | | | |
|  | Mozambique | No data identified | | | | | | |
|  | Niger | No data identified | | | | | | |
|  | Rwanda | No data identified | | | | | | |
|  | Sierra Leone | No data identified | | | | | | |
|  | Somalia | No data identified | | | | | | |
|  | South Sudan | No data identified | | | | | | |
|  | Sudan | No data identified | | | | | | |
|  | Syrian Arab Republic | No data identified | | | | | | |
|  | Tajikistan | No data identified | | | | | | |
|  | Togo | No data identified | | | | | | |
|  | Uganda | CECH, 2019 (46) | No data identified | No data identified | National DR framework | Agenda setting (Later stage) | No data identified | No data identified |
|  | Yemen, Rep. | IDF 2020 | No | N/A | No data identified | Agenda setting (Early stage) | No | No DESP |
| **LMIC (Lower-middle income)** (n=50) | Angola | No data identified | | | | | | |
|  | Algeria | IDF,2020 | Not sure | No information | No data identified | Agenda setting (Early stage) | Photographic screening | Partially implemented |
|  | Bangladesh | Muqit MMK, 2019 | Yes - DM health policy | No | Yes – Action Plan | Agenda Setting (Later stage) | Fundus screening (opportunistic) | Partially implemented (Sub-national) |
|  |  | IDF 2020 | No | N/A | No data identified | Agenda setting (Early stage) | Screening primarily by eye doctors in clinics. | Partially implemented (Regionally) |
|  | Benin | No data identified | | | | | | |
|  | Bhutan | No data identified | | | | | | |
|  | Bolivia | No data identified | | | | | | |
|  | Cape Verde | Schemann JF et al, 2006 (20) | No | No | Yes (National Eye Care Plan) | Agenda Setting (Later stage) | No information | No data identified |
|  | Cambodia | No data identified | | | | | | |
|  | Cameroon | IDF 2020 | Not sure | No information | Not sure | Agenda setting (Early stage) | No | No DESP |
|  | Comoros | No data identified | | | | | | |
|  | Congo, Rep. | IDF, 2020 | Yes (National DM Policy) | Yes (DR included in national DM policy) | No data identified | Policy formation | Photographic screening | Yes (National) |
|  | Côte d'Ivoire | No data identified | | | | | | |
|  | Djibouti | No data identified | | | | | | |
|  | Egypt | IDF,2020 | Yes (National DM Policy) | Not sure | No data identified | Agenda setting (Early stage) | No | No DESP |
|  | El Salvador | No data identified | | | | | | |
|  | Eswatini | No data identified | | | | | | |
|  | Ghana | CECH, 2019 (46) | No data identified | No data identified | National DR framework in place | Agenda setting (Later stage) | No data identified | No data identified |
|  | Honduras | IDF,2020 | Not sure | No information | Not sure | Agenda setting (Early stage) | Screening primarily by eye doctors in clinics | Partially implemented (Nationally) |
|  | India | Murthy GVS et al, 2020 (34) | No – NCD guidelines including DM prepared | No | No – DR guidelines (not legal documentation) | Agenda setting (Early stage) | Various modalities from static to mobile | Partially implemented (Sub-national) |
|  |  | IDF,2020 | Yes (National DM Policy) | No | No data identified | Agenda setting (Early stage) | No | No DESP |
|  | Kenya | Mwangi et al, 2018 (47) | No | No | No | Agenda setting (Early stage) | Opportunistic DES | Partially implemented (Sub-national) |
|  |  | Mwangi et al, 2020 (45) | Yes – Diabetes included in national health policy framework | No data identified | No data identified - clinical guidelines for DR available | Agenda setting (Later stage) | No data identified | Partially implemented  (Sub-national) |
|  |  | IDF,2020 | Yes (National DM Policy) | Yes (DR included in national DM policy) | No data identified | Policy formation | Photographic screening | Partially implemented (Regionally) |
|  | Kiribati | Clark M, White E, 2019 (8) | Yes –DM included in NCD Strategic Plan 2016-2019. | DR included as health indicator. | Yes - National Eye Care Strategy Plan 2015-2018, | Policy Formation | DR programme is coordinated by eye health nurses who are employed by the government and, therefore, fully integrated within government | No DESP |
|  | Kyrgyzstan (Kyrgyz Republic) | Mueller B et al, 2020 (15) | No | No | New initiatives introduced by MOH to put eye health, including DR on agenda. | Agenda setting (Early stage) | No | No DESP |
|  | Lao PDR (Laos) | No data identified | | | | | | |
|  | Lesotho | IDF,2020 | No | N/A | No data identified | Agenda setting (Early stage) | Screening primarily by eye doctors in clinics | Partially implemented (Sub-national) |
|  | Mauritania | No data identified | | | | | | |
|  | Micronesia, Fed. Sts. | No data identified | | | | | | |
|  | Moldova | No data identified | | | | | | |
|  | Mongolia | IDF,2020, | No | N/A | No information | Agenda setting (Early stage) | Screening primarily by eye doctors in clinics | Partially implemented (Regionally) |
|  | Morocco | No data identified | | | | | | |
|  | Myanmar | No data identified | | | | | | |
|  | Nepal | Upreti SR et al, 2016 (41) | Yes –NCD Multisectoral Action Plan (2014-2020 | No | No | Agenda setting (Early stage) | At public hospitals, DR services are available at zonal, regional and tertiary-level health care centres. | Partially implemented (zonal, regional and tertiary level) |
|  | Nicaragua | No data identified | | | | | | |
|  | Nigeria | IDF,2020, | Not sure | No information | Not sure |  | No data identified | No data identified |
|  |  | CECH, 2019 (46) | No data identified | Developing national DR framework | Developing national DR framework | Agenda setting (Later stage) | No data identified | No data identified |
|  | Pakistan | Jawa A et al, 2016 (17) | No | No | No | Agenda setting (Early stage) | Integrated primary care model (optometrists examined patients with ophthalmoscope) | Partially implemented (Sub-national) |
|  |  | Shah SP et al, 2008 (25) | No | No | No | Agenda setting (Early stage) | No data identified | No data identified |
|  | Papua New Guinea | Burnett A et al, 2019 (RAAB) (22) | No information | No data identified | No data identified | Agenda setting (Early stage) | No data identified | No data identified |
|  | Philippines | Beran D, Higuchi M, 2013 (43) | Yes – (National Objectives for Health 2005-2010) | No data identified | No data identified | Agenda setting (Early stage) | No data identified | No data identified |
|  |  | IDF,2020, | No | N/A | No data identified | Agenda setting (Early stage) | No | No DESP |
|  | São Tomé and Principe | No data identified | | | | | | |
|  | Senegal | No data identified | | | | | | |
|  | Solomon Islands | Clark M, White E, 2019 (8) | Yes - National Health Strategic Plan 2016-2020 identifies NCD, including DM, as a key priority for action | No | Yes - The National Eye Care Strategic 2015-2019 | Policy formation | Outreach screening | Partially implemented (Sub-national) |
|  | Sri Lanka | IDF, 2020 | Not sure | No data identified | No data identified | Agenda Setting (Early stage) | Photographic screening | Partially implemented (Regionally) |
|  | Tanzania | Cleland CR et al, 2016 (32) | No | No | No | Agenda setting (Early stage) | Integrated clinic-based mobile retinal screening) | Partially implemented (Sub-national) |
|  |  | CECH, 2019 (46) |  |  | National DR framework in place | Agenda setting (Later stage) |  |  |
|  | Timor-Leste | Sugnanam KK et al, 2016 (38) | No | No | No | Agenda setting (Early stage) | No data identified | No data identified |
|  | Tunisia | IDF,2020 | Yes (National DM Policy) | Yes (DR included in national DM policy) | No data identified | Policy formation | Photographic screening | Partially implemented (nationally) |
|  | Ukraine | No data identified | | | | | | |
|  | Uzbekistan | IDF,2020 | Yes (National DM Policy) | Yes (DR included in national DM policy) | No data identified | Policy formation | Photographic screening | Partially implemented (Locally) |
|  | Vanuatu | Clark M, White, 2019 (8) | Yes (Health Sector Strategy 2017-2020) | Yes - The NCD Policy and Strategic Plan 2016-2020 has a specific strategic objective on eye health, including DR. | No | Policy Formation | Outreach screening | Partially implemented (Sub-national) |
|  | Vietnam | Beran D, Higuchi M, 2013 (43) | Yes (Preliminary National Plan for DM) | No data identified | No data identified | Agenda setting (Early stage) | Photographic Screening | Partially implemented (Sub-national) |
|  | West Bank and Gaza (Palestine) | IDF, 2020 | Yes | Yes | No data identified | Policy formation | Photographic screening | Partially implemented (locally) |
|  | Zambia | Lewis AD, 2018 | Yes – NCDs including DM included in National Health Strategy Plan. | No | No | Agenda setting (Early stage) | Mobile DR screening | Partially implemented (Sub-national) |
|  |  | IDF, 2020 | No | N/A | No data identified | Agenda setting (Early stage) | No | No DESP |
|  | Zimbabwe | No data identified | | | | | | |
| **UMIC (Upper middle income countries)**  (n=58) | Albania | IDF, 2020 | No | N/A | No data identified | Agenda setting (Early stage) | No | No DESP |
|  | American Samoa | No data identified | | | | | | |
|  | Argentina | Caporale JE et al, 2013 (36) | No (National DM guidelines) | No | No | Agenda setting (Early stage) | No | No DESP |
|  |  | IDF,2020 | Yes (National DM Policy) | Not sure | No data identified | Agenda setting (Early stage) | Not sure | No data identified |
|  | Armenia | No data identified | | | | | | |
|  | Azerbaijan | No data identified | | | | | | |
|  | Belarus | No data identified | | | | | | |
|  | Belize | IDF,2020 | Not sure | No data identified | No data identified | Agenda setting (Early stage) | No | No DESP |
|  | Bosnia and Herzegovina | IDF,2020 | Not sure | No data identified | No data identified | Agenda setting (Early stage) | Screening primarily by eye doctors in clinics | Partially implemented (Sub-national) |
|  | Botswana | Blake AM et al, year, 2015 (21) | No data identified | No data identified | No data identified | No data identified | Photographic screening by ophthalmic nurses | Yes – National DESP |
|  |  | CECH, 2019 (46) | No data identified | No data identified | Yes – National DR framework in place | Agenda Setting (Later stage) | No data identified |  |
|  | Brazil | IDF,2020, | Yes (National DM Policy) | Yes (DR included in national DM policy) | No data identified | Policy formation | Photographic screening | Yes – National DESP |
|  | Bulgaria | No data identified | | | | | | |
|  | China | Peng J et al, 2011 (18) | No | No | No | Agenda setting (Early stage) | Community DR telehealth system (Dilated fundoscopy) | Partially implemented (Sub-national) |
|  | Colombia | No data identified | | | | | | |
|  | Costa Rica | Castellon RIA, 2019 (RAAB) (16) | No | No | Developing an Eye Healthcare National Programme inc DR (Action Plan) | Agenda Setting (Later stage) | No data identified | No data identified |
|  | Cuba | No data identified | | | | | | |
|  | Dominica | No data identified | | | | | | |
|  | Dominican Republic | No data identified | | | | | | |
|  | Equatorial Guinea | No data identified | | | | | | |
|  | Ecuador | IDF, 2020 | No | N/A | No data identified | Agenda setting (Early stage) | Not sure | Partially implemented (Locally) |
|  | Fiji | Clark M, White E, 2019 (8) | Yes ( National Strategic NCD Plan) | No | No | Agenda setting (Early stage) | No data identified | No data identified |
|  |  | Damato EM et al, 2014 (39) | No | No | No | Agenda setting (Early stage) | Undilated fundus photography at Pacific Eye Institute | Partially implemented (Locally) |
|  | Gabon | No data identified | | | | | | |
|  | Georgia | No data identified | | | | | | |
|  | Grenada | No data identified | | | | | | |
|  | Guatemala | Chávez GMG et al, 2015 | No | No | No | Agenda setting (Early stage) | No data identified | No data identified |
|  | Guyana | No data identified | | | | | | |
|  | Indonesia | No data identified | | | | | | |
|  | Iran, Islamic Rep. | Katibeh M et al, 2017 (31) | No | No | No | Agenda setting (Early stage) | No | No data identified |
|  | Iraq | IDF, 2020 | Not sure | No data identified | No data identified | Agenda setting (Early stage) | No | No DESP |
|  | Jamaica | CECH, 2019 (46) | No data identified | No data identified | National DR framework in place | Agenda setting (Later stage) | No data identified | No data identified |
|  | Jordan | No data identified | | | | | | |
|  | Kazakhstan | No data identified | | | | | | |
|  | Kosovo | No data identified | | | | | | |
|  | Lebanon | IDF, 2020 | Not sure | No data identified | No data identified | Agenda setting (Early stage) | Photographic screening | Partially implemented (Regionally) |
|  | Libya | No data identified | | | | | | |
|  | Malaysia | IDF, 2020 | Yes (National DM Policy) | Yes (DR included in national DM policy) | No data identified | Policy formation | Photographic screening | Yes – National DESP |
|  | Maldives | No data identified | | | | | | |
|  | Marshall Islands | No data identified | | | | | | |
|  | Mauritius | IDF, 2020 | No | N/A | No information | Agenda setting (Early stage) | Photographic screening | Yes - National DESP |
|  | Mexico | Polack S et al, 2012 (33) | No | No | No | Agenda setting (Early stage) | No | No DESP |
|  |  | Barquera S et al, 2013 (37) | Yes – National DM action programme. NCDs included in National Health Plan | No | No | Agenda setting (Early stage) | No data identified | No data identified |
|  |  | IDF 2020 | Yes (National DM Policy) | No | No data identified | Agenda setting (Early stage) | No data identified | No data identified |
|  |  | Lopez-Star EM et al, 2018 (26) | No data identified | No data identified | No data identified | Agenda setting (Early stage) | No data identified | No DESP |
|  | Montenegro | No data identified | | | | | | |
|  | Namibia | No data identified | | | | | | |
|  | North Macedonia | IDF, 2020 | Yes (National DM Policy) | Yes (DR included in national DM policy) | No data identified | Policy formation | Screening primarily by eye doctors in clinics | Partially implemented (Regionally) |
|  | Nauru | No data identified | | | | | | |
|  | Paraguay | No data identified | | | | | | |
|  | Peru | Salamanca O et al, 2018 (29) | Yes - Peru's concerted national health plan 2007–2020 provided technical advice at the national level and identified DM as a priority for the health agenda. | No | No | Agenda setting (Early stage) | Non-mydriatic screening (secondary and tertiary level hospital) | Partially implemented (Sub-national) |
|  |  | IDF, 2020 | Yes (National DM Policy) | Not sure | No data identified | Agenda setting (Early stage) | No | No DESP |
|  | Romania | No data identified | | | | | | |
|  | Russian Federation | No data identified | | | | | | |
|  | Samoa | Clark M, White E, 2019 (8) | No - No current strategic plan although a draft NCD plan is in development | No | No | Agenda setting (Early stage) | Outreach screening (binocular indirect-ophthalmoscope) | Partially implemented (Sub-national) |
|  | Serbia | IDF, 2020 | Yes (National DM Policy) | Yes (DR included in national DM policy) | No data identified | Policy formation | Screening primarily by eye doctors in clinics | Yes – National DESP |
|  | South Africa | Cook S et al, 2013 | No | No | No | Agenda setting (Early stage) | Opportunistic model | Partially implemented (National DESP in planning) |
|  |  | Cook S et al, 2014 (24) | No | No | No | Agenda setting (Early stage) | Photographic screening- opportunistic basis. | Partially implemented (Developed framework for national DR screening) |
|  | St. Lucia | IDF, 2020 | No | N/A | No data identified | Agenda setting (Early stage) | Photographic screening | Partially implemented (Regionally) |
|  | St. Vincent and the Grenadines | No data identified | | | | | | |
|  | Suriname | No data identified | | | | | | |
|  | Thailand | Reutrakul S et al, 2016 (35) | Yes – Thailand Healthy Lifestyle Strategic Plan | No data identified | No data identified | Agenda setting (Early stage) | Mobile Eye Clinics | Partially implemented |
|  |  | Isipradit S et al, 2014 (RAAB) (27) | No | No | Yes – (Designated VI as national health policy, including DR) | Policy formation | No data identified | No data identified |
|  |  | Chetthakul T et al, 2006 (19) | No | No | No | Agenda setting (Early stage) | Mobile eye clinics | Partially implemented (Sub-national) |
|  | Tonga | Clark M, White E, 2019 (8) | Yes - National Health Strategic Plan 2015-2020 identifies NCD as a key priority. | No | No | Agenda setting (Early stage) | No data identified | No data identified |
|  | Turkey | Kilic B et al, 2014 (42) | Yes – Strategic Plan | No | No | Agenda setting (Early stage) | No data identified | No data identified |
|  | Turkmenistan | No data identified | | | | | | |
|  | Tuvalu | No data identified | | | | | | |
|  | Venezuela, RB | No data identified | | | | | | |
| Policy cycle consists of 4 main stages: (1) agenda setting is divided into an early stage (situational analysis) and late stages (action plans), (2) policy formation, (3) implementation and, (4) evaluation  National DESP implementation, Red = No DESP, Yellow= partially implemented (sub-national, regional), Green = national DESP available  Regionally = DESPs are implemented in different regions in the country (not a national level)  *If more than one resource was obtained for a single country, the most positive finding was applied to the table. | | | | | | | | |
